# Identification and Validation of Genetic Variations in Transgenic Chinese Cabbage Plants (*Brassica rapa* ssp. *pekinensis*) by Next-Generation Sequencing

**DOI:** 10.3390/genes12050621

**Published:** 2021-04-22

**Authors:** So-Jeong Kim, Jee-Soo Park, Yun-Hee Shin, Young-Doo Park

**Affiliations:** Department of Horticultural Biotechnology, Kyung Hee University, 1732 Deogyoung-daero, Giheung-gu, Yongin-si, Gyeonggi-do 17104, Korea; coalla110@naver.com (S.-J.K.); jeesoo_92@naver.com (J.-S.P.); yunhee94@naver.com (Y.-H.S.)

**Keywords:** *Brassica rapa*, in vitro culture, resequencing, single nucleotide polymorphisms, transgenic plants

## Abstract

Transgenic plants are usually produced through tissue culture, which is an essential step in *Agrobacterium*-mediated plant transformation. However, genomic variations, termed somaclonal variations, have been detected in transgenic plants cultured in vitro. The occurrence of these variations should be as low as possible to secure the stability of transgenic crops. Determining the cause and mechanism of somaclonal variations in tissue culture-derived plants will help reduce the rate of variation and promote the stable expression of genes in transgenic plants. In order to determine the genetic variability in transgenic Chinese cabbage plants, we performed whole-genome resequencing and compared the sequencing data with the ‘CT001’ reference genome. The variation candidates that were expected to consistently occur in the transgenic lines were selected and validated. The single nucleotide polymorphism (SNP) and insertion and deletion (InDel) candidates were identified using the resequencing data and validated by reverse transcription (RT)-PCR analysis. The deduced amino acid sequences were used to determine whether the variations caused changes in the resulting polypeptide, and the annotations of the mutated genes were analyzed to predict the possible effects of the SNPs on gene function. In conclusion, we selected and validated the genetic variations identified in transgenic Chinese cabbage plants. Their genomes were expected to be affected by the process of *Agrobacterium*-mediated transformation. The findings of our study will provide a genetic basis for transgenic plant research.

## 1. Introduction

Plant tissue culture is a fundamental plant biotechnology tool employed in the production of large numbers of genetically identical plantlets. This in vitro technology is widely applied in various research areas, such as the mass production of secondary metabolites and genetic transformation of plants, for crop improvement. Plant cells are cultured under sterile conditions and often undergo dedifferentiation based on their totipotency. In the production of transgenic plants, the genetic stability of a produced plant and its gene expression is the most important issue.

Unfortunately, some variations, called somaclonal variations, can be observed in plants produced in vitro by regenerating somatic cells [1,2]. These variations can, thus, cause problems with the genetic uniformity of the transgenic plants and lead to subsequent negative effects on the use of genetic transformation as well as tissue culture. Therefore, the detection of somaclonal variations in tissue culture-derived plants is essential for conserving elite varieties and avoiding undesired characters. Many studies have used whole-genome sequencing to identify genomic variations in transgenic [3,4] and CRISPR/Cas9-edited plants [5,6].

Genetic variations have been observed in *Arabidopsis thaliana* transgenic plants [7]. The development of next-generation sequencing (NGS) technology has enabled the effective analysis of genome-wide DNA polymorphisms such as single nucleotide polymorphisms (SNPs) and insertions and deletions (InDels) [8]. Consequently, the cost of analysis and marker development has been reduced [9]. Whole-genome resequencing is a reliable approach used to identify sequence mutations [10,11,12].

NGS has also allowed the characterization of genome-wide DNA methylation patterns on a large scale and at single-base resolution, including whole-genome bisulfite sequencing (WGBS), small RNA sequencing, and chromatin immunoprecipitation sequencing (ChIP-seq) [13].

Chinese cabbage (*Brassica rapa* ssp. *pekinensis*) is one of the most important vegetables worldwide. The reference genome of the *B. rapa* variety Chiifu-401-42 has been published in 2011 [14], and the pseudomolecule genome of the inbred line ‘CT001’ has been constructed for precise genome research [15].

In this study, whole genome resequencing was performed in transgenic Chinese cabbage plants. Genetic variations, including SNPs and InDels, were identified, and the mutation candidates expected to commonly occur in transgenic plants were selected and analyzed. The present study is expected to provide an overview of the genetic variations of transgenic plants.

## 2. Materials and Methods

### 2.1. Construction of Transgenic Lines

**Figure 1:**
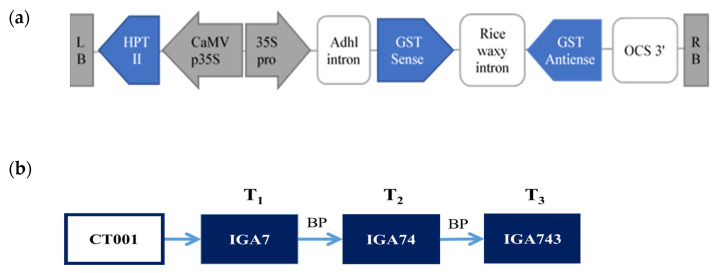
Schematic map of the T-DNA region of the glutathione S-transferase (GST) RNAi binary vector used for Chinese cabbage transformation (**a**) and strategy for construction of the progeny lines of transgenic Chinese cabbage plant, IGA7 (**b**). LB, left border; HPT II, hygromycin resistant gene; CAMV35S and 35S pro, cauliflower mosaic virus 35S promoter; Adhl intron, intron of the alcohol dehydfogenase-7 (Adhl) gene; GST sense, glutathione S-transferase gene; OCS-3′, The 3′-flanking region of octopine synthase gene; RB, right border.

For identifying somaclonal variations in transgenic plants, IGA transgenic lines were used in which the glutathione S-transferase (*GST*) gene was downregulated using an RNA interference (RNAi) vector (Figure 1a). The IGA lines were identified with a single copy of the T-DNA and were advanced up to the T_3_ generation by bud pollination. For preparing experimental materials, seven seeds of each generation were sown in the greenhouse of Kyung Hee University, Yongin, Korea. The hygromycin-selected transgenic plants were transplanted in the greenhouse of Hanguk Jongmyo (Pyeongtaek, Korea), and bud pollination was performed. The T_2_, and T_3_ lines were also produced as described above (Figure 1b) and then used for resequencing.

### 2.2. Genomic DNA Extraction and Confirmation of Transgenic Plants.

Young leaves of the transgenic plants were finely ground in a mortar using liquid nitrogen, and genomic DNA was extracted by modifying the method of Dellaporta et al. [16]. The quality and quantity of the extracted genomic DNA were checked using the Trinean DropSense instrument (Trinean, Gentbrugge, Belgium) and the PicoGreen assay (Molecular Probes, Eugene, OR, USA).

PCR selection was performed on the hygromycin resistance gene, a selection marker of the GST RNA*i* vector used for transforming the IGA7 lines. To verify the T-DNA insertion of the transgenic plants, a PCR was performed with the genomic DNA using the following primers: HPT F: (5′–TTT CCA CTA TGC GCG AGT AC–3′) and HPT-R (5′–TGT CGA GAA GTT TCT GAT CGA–3′). The PCR amplification was performed in a total reaction volume of 20 μL using a thermocycler (Applied Biosystem, Carlsbad, CA, USA). The PCR program was composed of the following steps: initial denaturation at 94 °C for 1 min, 35 cycles of denaturation at 95 °C for 30 s, annealing at 37 °C for 30 s, and extension at 72 °C for 30 s, and final extension at 72 °C for 10 min. PCR products were resolved on 1.0% agarose gel in 1X TBE (Tris-borate-EDTA) buffer, stained with ethidium bromide and photographed under UV light. The intergenic genomic locations of the T-DNA inserted in the transgenic plants were analyzed using a web tool, flanking sequence tag validator (FSTVAL; http://bioinfo.mju.ac.kr/fstval/; accessed on 21 July 2020) [17].

### 2.3. Chromosome Number Analysis of Transgenic Plants

The seeds of ‘CT001’, IGA7, IGA74, and IGA743 were germinated on petri dishes at 25 °C for 48 h. After germination, root tips were collected and incubated in 8-hydroxyquinoline solution at 20 °C for 4 h. Subsequently, the root tips were fixed in acetic acid:ethanol (1:3) fixative solution overnight and stored in 70% ethanol at −20 °C. The chromosome preparations were made by squashing the root tips. The squashed root tips were then treated with an enzyme mixture (pectolyase (0.3%), cytohelicase (0.3%), and cellulase (0.3%) in 150 mM citrate buffer) for 1 h at 37 °C. Thereafter, the root tips were squashed in a drop of 60% acetic acid, followed by air drying. The chromosomes were counter-stained with 1 μL·mL^−1^ of 4′,6-diamidino-2-phenylindole (DAPI) in 100 μL Vecta-shield (Vector laboratories Inc., Burlingame, CA, USA) and observed under a fluorescent microscope (Olympus BX 61, New York, NY, USA). Images were taken using a charge-coupled device (CCD) and then processed using the Genus Imaging System (Applied Imaging Corporation, Genus version 3.8 program, Foster City, CA, USA). To increase the reliability, the number of chromosomes was counted three times for each line.

### 2.4. Sequencing and Mapping

The verified transgenic plants, IGA7, and IGA743, were used for genetic variation analysis. The quality and quantity of each DNA sample were checked using the PicoGreen assay. The genomic DNA of each transgenic line was fragmented using the Covaris system (Covaris, Woburn, MA, USA). Sequencing libraries with 300~400 bp insert sizes were constructed using the Illumina TruSeq DNA Nano Kit (Illumina, San Diego, CA, USA) according to the manufacturer’s instructions. The quality of raw sequencing reads was checked using the BioanalyzerDNA 1000 chip (Agilent Technologies, Santa Clara, CA, USA). Paired-end resequencing reads were generated by using the Illumina HiSeq X System (Illumina) according to the manufacturer’s instructions.

Raw data were cleaned by removing adaptor contamination and low-quality reads before further analysis using the Trimmomatic program [18]. The trimmed paired reads of each accession were mapped to the ‘CT001’ pseudomolecule genome [15] using the Burrows-Wheeler Aligner (BWA-MEM version 0.7.17-r1188) [19] with the default settings. The read grouping and removal of PCR duplicates were carried out using Picard 1.112 (http://broadinstitute.github.io/picard/accessed on 16 November 2020), and the misalignments caused by InDels were corrected by local re-alignment using the Genome Analysis Toolkit (GATK).

### 2.5. Discovery of SNPs and InDels from Resequencing Genome Data

Variant calling from the resequencing data of non-transgenic and transgenic plants was performed by alignment to the ‘CT001’ pseudomolecules. We applied GATK HaplotypeCaller [20] to call genetic variants in transgenic plants. The SNPs with low read depth (DP < 10) and low genotype quality (GQ < 30) were excluded from further analysis. The candidate SNPs specific for the transgenic plants were called, and the genomic positions of the SNPs and InDels within the aligned reads were identified and compared. The SNPs identified and filtered in each accession were merged and compared with each other to identify the promising SNPs specific for the transgenic plants. From the raw SNPs and InDels, we selected the variation candidates that were identified in both T_1_ and T_3_ progenies of transgenic plants, when compared to a non-transgenic plant. If the reads were not properly mapped even in one line, they were filtered out. These variation candidates were considered as the genetic variations that might have occurred as a result of the plant transformation process and persisted in the progenies.

The positions of the genetic variants on the genes were confirmed through the custom scripts and depicted on the chromosomes using the web tool MapGene2Chromosome2 (http://mg2c.iask.in/mg2c_v2.1/; accessed on 4 December 2020) [21].

### 2.6. RT-PCR Analyses of the Selected Exonic SNPs

RT-PCR analysis was used to confirm the mutated sequences using the complementary DNAs of the transgenic plants. For the RT-PCR analysis, primer sets were designed using the Vector NTI^®^ program (Invitrogen, Carlsbad, CA, USA), using flanking sequences of the exonic SNPs identified in transgenic plants (Table S1). RT-PCR were performed using thermocycler (Applied Biosystem) with Maxime™ PCR PreMix Kit (iNtRON, Seongnam, Korea) in a total 20 μL reaction volume. The amplification program was as follows: an initial denaturation of 5 min at 95 °C was followed by 35 cycles of 30 s at 95 °C, 30 s at 60 °C, and 30 s at 72 °C, and a final extension of 10 min at 72 °C. The obtained RT-PCR products were separated on 1.0% agarose gels by electrophoresis and eluted from the agarose gel. The eluted product was ligated with the pGEM-T easy vector (Promega, Madison, WI, USA) and sequenced (Macrogen Co., Seoul, Korea).

## 3. Results

### 3.1. Confirmation of Transgenic Plants

For the genomic analysis of the transgenic Chinese cabbage plants, we used the T_1_ and T_3_ generations of the IGA transgenic plants with down-regulated expression of the GST gene. A PCR analysis was performed to confirm the presence of the inserted T-DNA using the genomic DNA of the transgenic plants and a set of primers targeting the hygromycin resistance gene with an expected size of 1149 bp. The amplified products were detected by agarose gel electrophoresis, and the insertion of T-DNA fragments was confirmed.

**Figure 2:**
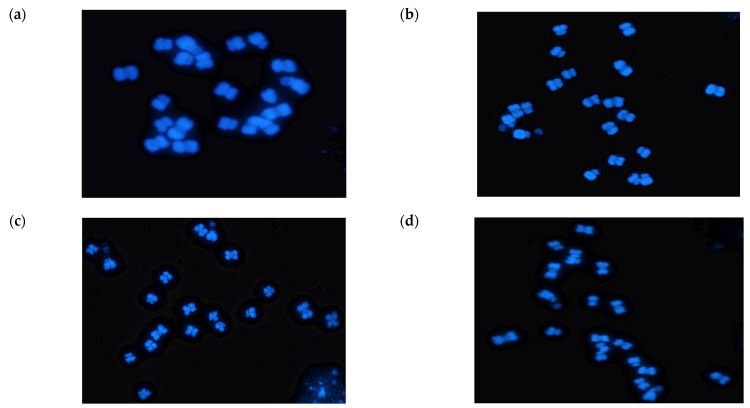
Analysis of chromosome number of non-transgenic and transgenic Chinese cabbage plants. (**a**) Non-transgenic control ‘CT001’ line (**b**) IGA7 (**c**) IGA74 (**d**) IGA743. The number of chromosomes in both non-transgenic control and transgenic plants was found to be 20.

**Figure 3:**
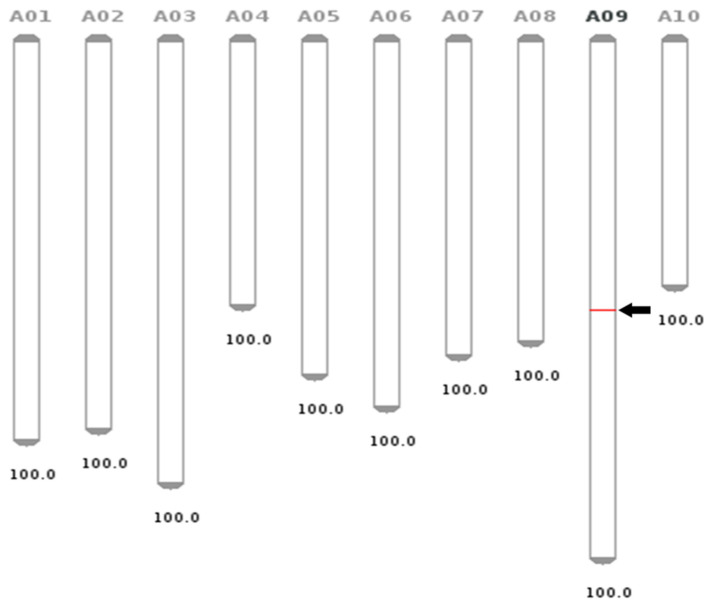
Detection of T-DNA insertion site in IGA7 by FSTVAL analysis. Insertion position (red bar indicated by arrow) in chromosome of the IGA7 was intergenic region on chromosome 9. Each number under the chromosome means the drawing scale (kb/px). The analysis suggested that the T-DNA was located 4.286 kb upstream of the Bra028059 gene that encodes an N-terminal-domain-containing essential protein Yae1 on chromosome 9.

As a result of chromosome analysis, 20 chromosomes were observed both in the transgenic and non-transgenic control plants (Figure 2). Additionally, the flanking sequence of the transgene was confirmed using the web tool FSTVAL. The analysis suggested that the insert was located 4.248 kb upstream of the Bra028059 gene that encodes an N-terminal-domain-containing essential protein Yae1 on chromosome 9 (Figure 3). The genomic DNAs of the confirmed transgenic plants were analyzed for genetic variation.

### 3.2. Sequencing and Trimming

The whole genomes of the T_1_ (IGA7) and T_3_ (IGA743) progeny lines of the transgenic plants were resequenced using the Illumina Hiseq X platform. A sequencing library was generated using the TruSeq Nano Kit (Illumina), and the library quality control (QC) was analyzed. The selected plant libraries with an average size of 420 bp were resequenced using the Illumina HiSeq X platform. Approximately 34 million raw reads, amounting to a total of approximately 5.2 Gb data, were generated (Table S2). After trimming, an average sequence of 4.3 Gb remained as high-quality reads, ranging from 31.3 to 31.7 million reads per accession. Of the trimmed sequence reads, approximately 80% of raw data were aligned to the ‘CT001’ reference genome with an average sequencing depth of 11X (Table S3). On average, approximately 15.6 million paired-end reads were aligned to the ‘CT001’ reference genome and showed 97~98% genome coverage.

### 3.3. Discovery of Genetic Variations in T_1_ and T_3_ Transgenic Plants

**Figure 4:**
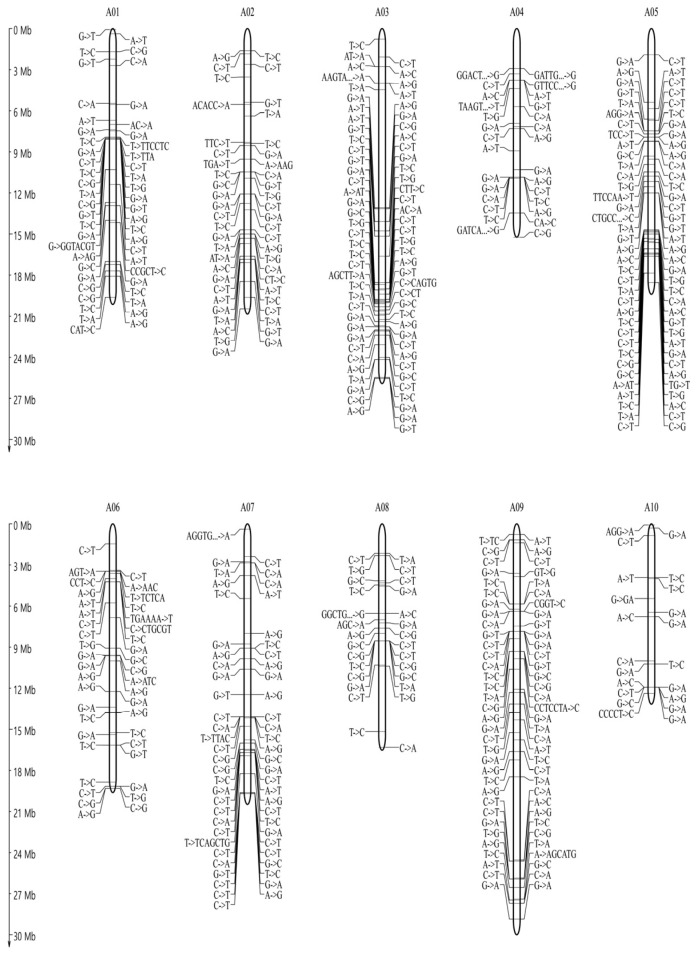
Chromosomal location of detected SNP and InDel in the transgenic Chinese cabbage plants. A total of 3294 SNP and InDel candidates occurring in both T_1_ and T_3_ transgenic plants were identified. The SNP and InDel candidates were distributed throughout the entire chromosome.

**Figure 5:**
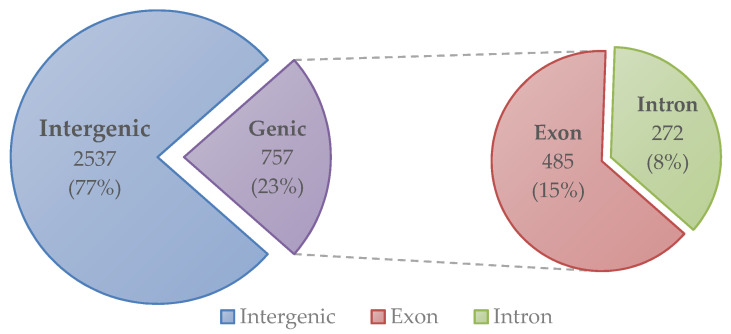
Number and percentage of SNP and Indel occurring in both T_1_ and T_3_ transgenic Chinese cabbage plants located in genic and intergenic regions.

To determine the genetic variations, which occurred during the plant transformation process and was maintained in the progenies, the genome of the T_1_ and T_3_ transgenic plants were analyzed. The resequencing data of the IGA7 and IGA743 transgenic plants were compared to the reference sequences to determine if the SNP and InDel candidates were expected to consistently occur in transgenic plants. The presence of SNPs or InDels was excluded if they were not mapped in the data of either IGA7 or IGA743. As a result, a total of 3294 SNP and InDel candidates occurring in both T_1_ and T_3_ transgenic plants were identified (Table 1). Our findings revealed the variation candidates that consistently occurred in the transgenic plants and were maintained in the progeny lines. Furthermore, the distribution of the SNPs and InDels occurring in all transgenic plants within the genome of B. rapa ssp. pekinensis ‘CT001’ was investigated, and it was found that each variation was distributed throughout the entire chromosome (Figure 4). Among the selected variation candidates, 2753 were substitutions and 541 were InDels. It was observed that most (2537 SNP and InDel) of the variations occurred in intergenic regions, 485 in exons, and 272 in introns (Table 1 and Figure 5).

**Figure 6:**
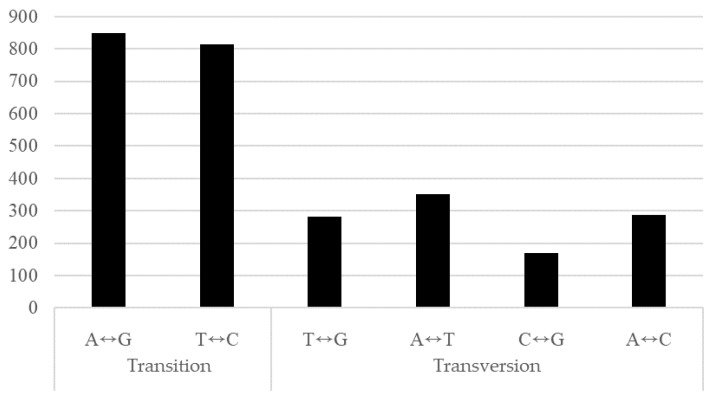
Analysis of substitutions which have been consistently identified in T_1_ and T_3_ transgenic Chinese cabbage plants.

Among the 2753 substitutions, all types of base changes were detected in the transgenic plants, but transition mutations, including both interchanges of purines (A-G) and pyrimidines (C-T), were greater than transversions, which are interchanges between purine and pyrimidine bases (Figure 6 and Table 2).

### 3.4. Analysis of Genes with SNP or InDel in Transgenic Plants

To analyze the functions of the genes associated with the SNPs and InDels consistently identified in the T_1_ and T_3_ generations of transgenic plants, functional annotation clustering was performed with the corresponding The Arabidopsis Information Resource (TAIR) IDs of the genes using the DAVID bioinformatics resource (https://david.ncifcrf.gov/; accessed on 11 January 2021) (Table 3). As a result, 33 plasma membrane genes clustered with the highest enrichment scores were selected.

### 3.5. Validation of the Exonic SNPs or InDels Identified in the Transgenic Plants and Their Progenies

**Figure 7:**
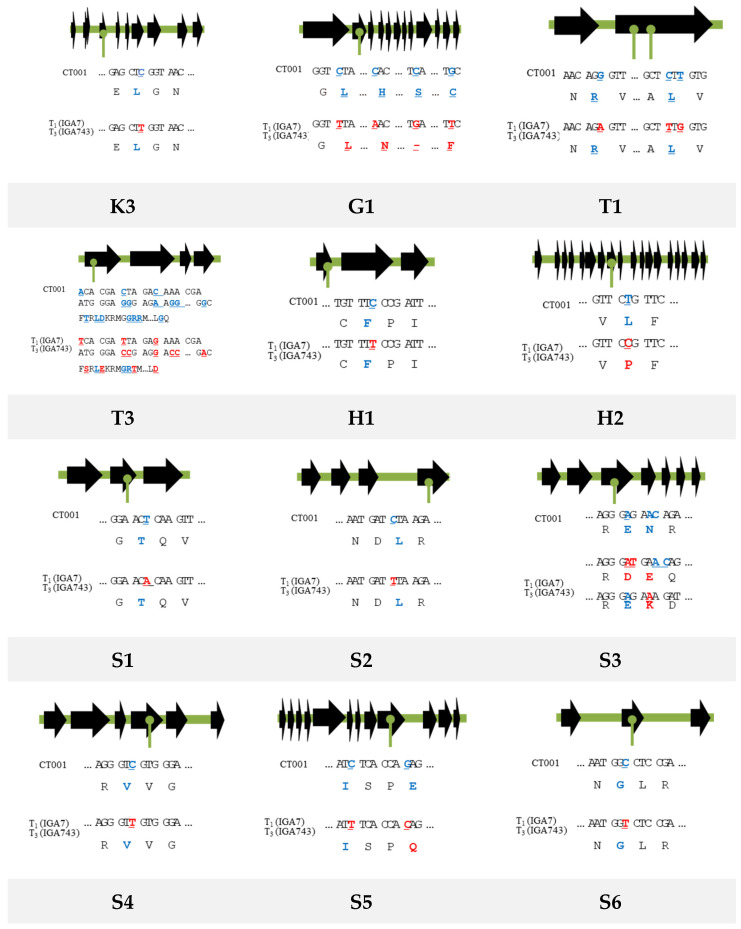
Nucleic acid and amino acid sequence analysis of the RT-PCR products of the target SNPs from non-transgenic and transgenic Chinese cabbage plants. Black arrows indicate exons of each gene and their coding directions. Green lines with a circle indicate the location with genomic variation. Original sequences are in blue and the altered sequences are in red.

For validation of the identified exonic SNPs or InDels in genome, RT-PCR of the 12 selected genes was conducted using cDNA synthesized from total RNA extracted from each transgenic line and primer sets for the selected genes. Among the genes consistently identified in the T_1_ and T_3_ transgenic plants, a total of 12 genes related to the plasma membrane and stress response were selected, and 12 sets of primers targeting each SNP were designed (Table S1). The primer set for K3 targets a kinase-related gene, G1 targets a transcription factor-related gene, S1 to S6 target stress response-related genes, and H1 and H2 target SNPs generated from the microtubule-related genes. An RT-PCR was conducted using the designed primer sets, and the amplified products produced the bands of expected sizes. The RT-PCR amplicons were extracted from the agarose gel and were sequenced to validate the existence of SNPs and InDels in the genome of the transgenic plants. Of the 12 primer sets designed for selected genes, one SNP was identified from seven primer sets, and two or more SNPs and InDel were identified in five primer sets (Figure 7). The positions of this SNP and InDel were also confirmed. Additionally, the deduced amino acid sequences of the RT-PCR amplicons were compared (Figure 7). Among the exonic SNP candidates, seven were found to be synonymous mutations that resulted in no changes in the amino acid; however, five mutations were non-synonymous mutations that altered the amino acid compositions.

In addition, the function of the genes with the exonic SNP or InDel in the IGA7 and IGA743 was also predicted (Table 4).

## 4. Conclusions

Transgenic technology, which is mainly based on plant tissue culture, including *Agrobacterium*-mediated plant transformation, is widely used to improve agronomic traits. However, unintended variations have been reported in tissue-cultured plants, including the transgenic plants that require stable transgene expression and phenotype. In this study, resequencing and variant calling were conducted using the non-transgenic and transgenic plants of Chinese cabbage (*B. rapa* ssp. *pekinensis* ‘CT001’). The genetic variations identified consistently in the T_1_ and T_3_ progeny lines of transgenic plants were analyzed. After gene clustering of the genes containing SNPs, a total of 12 SNPs with gene annotations of interest were selected and validated by RT-PCR methods. The findings in this study are expected to provide basic information for understanding genetic diversity in transgenic plants.

## 5. Discussion

Somaclonal variations have been reported in various tissue culture-derived plants, and the sources of the phenotypic variations of regenerants have been suggested to be genetic variation [5,22], epigenetic changes [23], chromosomal rearrangement [24], and aneuploidy [25].

We conducted whole-genome resequencing of the genomic DNA of non-transgenic and transgenic Chinese cabbage plants to analyze the genetic variation associated with the unintended somaclonal variation produced by in vitro culture and transformation procedures.

Before the resequencing analysis, the chromosome number of non-transgenic and transgenic Chinese cabbage plants used for analysis was determined in order to exclude possible variations due to abnormal chromosome numbers. As chromosomal abnormalities of regenerated or transgenic plants have been discovered in diverse plant species [26,27,28], chromosome numbers were counted in the root tips of all lines before further genomic analysis. For this analysis, transgenic plants were selected for chromosome number analysis; ‘CT001’, the control line used in *Agrobacterium*-mediated transformation of Chinese cabbage, and one individual for each of the T_1_, T_2_, and T_3_ generations of the IGA7 plants. As a result, 20 chromosomes were observed in both transgenic and non-transgenic control plants, thereby excluding the possibility of mutation due to chromosome number variation.

To determine the genetic variability in transgenic plants, whole genomes of the T_1_ (IGA7) and T_3_ (IGA743) progeny lines of the transgenic plants were resequenced and the resequencing data was compared with the ‘CT001’ reference genome sequences. A total of 3294 SNP and InDel candidates occurring in both T_1_ and T_3_ transgenic plants were detected and maintained despite the progress of the generation. The inheritance of some genetic variations in the progeny of the transgenic plant was similar to the results of genome analysis of regenerated rice [29,30]. When these variations were annotated against the ‘CT001’ reference genome, most of the variations (2537 SNPs and InDel) were observed to occur in the intergenic regions.

The distribution of the SNPs and InDels identified was uniformly distributed throughout the entire chromosome. The variation rate in the transgenic plants was estimated to be 7.49 × 10^−6^ bp/site/generation, which was higher than that of the spontaneous mutations in the sexually propagated lines of the Chinese cabbage inbred line ‘CT001’ (9.1 × 10^−9^ bp/site/generation) [15] and Arabidopsis (~7 × 10^−9^ bp/site/generation) [31]. It was also higher than the variation rate of the regenerated Arabidopsis (4.2 × 10^−7^~24.2 × 10^−7^ bp/site/generation) [22], rice (1.74 × 10^−6^ bp/site/generation) [30], and *B. rapa* ‘CT001’ plants (4.96 × 10^−7^ bp/site/generation) [32]. The transition:transversion ratio of the transgenic plants was 1.53, which was higher than that of the regenerated Arabidopsis (1.1) [30] but lower than that of the sexually propagated Arabidopsis (2.41) [31], rice (2.37) [29] and *B. rapa* (3.0) [15]. Compared to the ratio in the regenerated *B. rapa* ‘CT001’ plants (1.42) [32], the ratio detected in transgenic plants was slightly higher.

In the clustered result of genes in which SNPs and InDels were commonly identified in the transgenic plants IGA7 and IGA743 using DAVID, the enrichment score is a score for grouping based on the EASE score of each term member, and the higher the enrichment score, the more enriched. A group bound by the cluster enrichment score has similar biological meaning because they share similar gene members. In addition, the result of clustering is the summary of genes decided to be significant in relation to in vitro culture and transformation among those with a *p*-value less than 0.5.

The analysis of genes with variations in transgenic plants showed that 33 plasma membrane genes clustered with the highest enrichment scores. The plasma membrane is a membrane through which the T-DNA passes during *Agrobacterium*-based plant transformation [33], and it is predicted that SNPs might be generated in membrane-related genes because of the stress induced by T-DNA transfer or to promote T-DNA transfer. Furthermore, 19 genes involved in transport and four genes with transmembrane transport functions that may be involved in T-DNA delivery were identified. From the clustering result of these genes, it was predicted that variations in genes related to membrane or transmembrane migration may have occurred due to stress in the process of inserting T-DNA into nucleus in the cells during transformation in transgenic plants.

For further analysis, gene clustering was performed using the TAIR IDs of the 33 plasma membrane-related genes, which counted the highest number of genes among the clustered terms. As a result, the genes with functions related to transport and kinase activity were mainly clustered. Six genes functioning as stress responses were also clustered. As stress is expected to occur during the in vitro culture or plant transformation process [34], SNPs might have been generated in these genes to cope with stress in transformed plants.

Based on the results of the gene clustering analysis, 10 plasma membrane-related and stress-response genes were selected for further analysis. As a result of confirming the function of clustered genes in which SNP and InDel exist in the transgenic lines, K3 (CT001_A09315030) was predicted as a leucine-rich repeat (LRR) receptor-like serine/threonine-protein kinase and G1 (CT001_A02057850) was predicted as eukaryotic translation initiation factor 5B in *B. rapa*. LRR receptor-like serine/threonine-protein kinase is known to play important roles in regulating responses to various stresses, and previous studies have shown that it regulates environmental stress such as salinity stress and osmotic stress [35,36]. In this study, it can be explained that SNP in the stress-related gene, LRR receptor-like serine/threonine-protein kinase, occurred due to stress during the transformation process. The transport-related genes T1 (CT001_A06231480) and T3 (CT001_A08282960) were expected as inorganic phosphate transporter and S-type anion channel SLAH2, respectively. Among the stress-related genes S1~S6, S2 (CT001_A03117730, heat shock factor, HSF) increases the synthesis of special heat shock proteins (HSP) through a heat shock response (HSR), preventing stress-induced damage when cells are exposed to high temperatures. In addition to thermal stress, the HSF is also involved in tolerance to environmental stresses such as drought/desiccation stresses [37,38,39]. The gene function of S3 (CT001_A03119840) was identified as abscisic acid 8’-hydroxylase. Abscisic acid (ABA) is involved in numerous processes for the response to environmental stress, as well as for growth and development. Abscisic acid 8′-hydroxylase is a key enzyme to regulate ABA [40]. In *Arabidopsis thaliana*, ABA levels increase in salt, osmotic, and dehydration stress environments. At this time, it was confirmed that the expression of abscisic acid 8′-hydroxylase encoded genes was also increased to improve tolerance to these stresses [41]. It is believed that the occurrence of SNP or InDel in these stress-related genes occurs in response to stress resulting from the transformation process.

Among the genetic variations identified in the T_1_ and T_3_ transgenic plants, two SNPs classified as homozygous SNPs were also selected for further analysis. The H1 (CT001_A01012460) and H2 (CT001_A06202820) with SNPs were found to encode the RING-H2 finger protein ATL81 and the kinesin-like protein KIN-UA, respectively, which function as microtubule proteins. When plants are subjected to extracellular stimuli, such as environmental stresses, they phosphorylate tubulins and alter microtubule arrays to alter their cell structures, resulting in their ability to adapt to these stressful environments [42,43].

After RT-PCR analysis with each primer set located in the exon of the corresponding gene, we compared the deduced amino acid sequence of the RT-PCR amplicons. The amplicon with the expected product size was identified and revealed that alterations of the DNA sequence, to either a synonym or a nonsynonymous variation, can change the amino acid sequence. Seven base substitutions identified synonymous mutation candidates that did not cause changes in the amino acid. However, five were found to be non-synonymous mutations that alter the amino acid sequences of the polypeptide. In some cases, it was confirmed that more than one SNP appeared in the adjacent region of one gene, which was expressed asynchronously.

Base substitutions and InDels are the main genetic causes of somaclonal variations. This is because it can alter the amino acid sequence and lead to unintended phenotypic mutations. Many reports have confirmed that base substitution mutations alter the inferred amino acid sequence of proteins, resulting in phenotypic variations in regenerated plants [22,30].

In this study, although the genetic variations between non-transgenic and transgenic plants were confirmed, the difference in phenotype was not observed. Previous studies in rice [44] and maize [45] reported that most phenotypic variations were observed only in the parts of regenerated plants. Therefore, we should perform additional phenotypic analysis of transgenic plants to determine how the genetic variations in this study actually affect the phenotype.

In conclusion, in this study, we identified genetic variations such as SNPs and InDels that were consistently found in the transgenic plants and confirmed that some of these variations were maintained in the progeny lines. These genetic variations in transgenic lines could have been induced by in vitro culture and transformation procedures. The results of this study provide valuable resources for the genetic diversity in transgenic lines and help to improve our molecular understanding of somaclonal variation.

## Figures and Tables

**Table 1:** Number of the SNPs and InDels in each chromosome commonly identified T_1_ and T_3_ transgenic Chinese cabbage plants.

Chromosome.	Exon	Intron	Intergenic	Sum
A01	49	42	392	483
A02	49	41	266	356
A03	72	29	183	284
A04	28	13	147	188
A05	72	23	257	352
A06	41	20	289	350
A07	57	25	219	301
A08	28	27	236	291
A09	68	38	423	529
A10	21	14	125	160
Total	485	272	2537	3294

**Table 2:** Analysis of substitutions which have commonly occurred in T_1_ and T_3_ transgenic Chinese cabbage plants.

Occurred Region	Transition	Transversion
Exon	263	169
Intron	107	74
Intergenic	1295	845
Total variation	1665	1088

**Table 3:** Functional annotation clustering of genes with the exonic SNP or InDel in the T_1_ and T_3_ transgenic Chinese cabbage plants.

Cluster Enrichment Score	Category ^z^	Description	Count	*p* Value
1.14	GOTERM_CC_DIRECT	Plasma membrane	33	1.4 × 10^−2^
	UP_KEYWORDS	Transport	19	3.2 × 10^−2^
	UP_SEQ_FEATURE	Transmembrane region	19	3.2 × 10^−2^
	GOTERM_CC_DIRECT	Membrane	18	3.4 × 10^−2^
	UP_KEYWORDS	Membrane	49	4.4 × 10^−2^
	UP_KEYWORDS	Cell membrane	11	9.3 × 10^−2^
	UP_KEYWORDS	Transmembrane helix	40	1.2 × 10^−1^
	UP_KEYWORDS	Transmembrane	40	1.3 × 10^−1^
	GOTERM_BP_DIRECT	Transmembrane transport	4	1.5 × 10^−1^
	GOTERM_CC_DIRECT	Integral component of plasma membrane	4	2.2 × 10^−1^
	GOTERM_CC_DIRECT	Integral component of membrane	36	2.9 × 10^−1^
0.55	GOTERM_CC_DIRECT	Symporter activity	4	5.7 × 10^−3^
	UP_KEYWORDS	Symport	4	1.5 × 10^−2^
	UP_SEQ_FEATURE	Topological domain: Extracellular	8	2.9 × 10^−2^
	UP_KEYWORDS	Transport	19	3.2 × 10^−2^
	UP_SEQ_FEATURE	Transmembrane region	19	3.2 × 10^−2^
	UP_KEYWORDS	Nucleotide-binding	18	1.6 × 10^−1^
	UP_KEYWORDS	Glycoprotein	14	2.3 × 10^−1^
	GOTERM_BP_DIRECT	Transmembrane receptor protein tyrosine kinase signaling pathway	3	2.0 × 10^−1^
	GOTERM_MF_DIRECT	Protein serine/threonine kinase activity	8	2.1 × 10^−1^
	INTERPRO	Serine-threonine/tyrosine-protein kinase catalytic domain	5	2.2 × 10^−1^
	UP_KEYWORDS	Leucine-rich repeat	6	2.3 × 10^−1^
	INTERPRO	Leucine-rich repeat	4	3.9 × 10^−1^
0.5	GOTERM_CC_DIRECT	Membrane	18	3.4 × 10^−2^
	UP_SEQ_FEATURE	Transit peptide: Chloroplast	7	2.6 × 10^−1^

^z^: Original database or resource where the terms come from. GOTERM_CC, gene ontology term for cellular component; UP_KEYWORDS, keywords from UniProtKB; UP_SEQ_FEATURE, uniprot sequence feature; GOTERM_BP, gene ontology term for the description of biological process; GOTERM_MF, gene ontology term of molecular function; INTERPRO, terms from InterPro protein database.

**Table 4:** Description of genes with the exonic SNP and InDel in the T_1_ and T_3_ transgenic Chinese cabbage plants.

Name	Chr ^Z^	Position	Ref/Alt	Alteration	Location	Gene ID	Predicted Gene Description
K3	A09	6,785,472	C/T	substitution	Exon7	CT001_A09315030	PREDICTED: probable LRR receptor-like serine/threonine-protein kinase At1g63430 [Brassica rapa]
G1	A02	11,445,193	C/T	substitution	Exon11	CT001_A02057850	PREDICTED: eukaryotic translation initiation factor 5B [Brassica rapa]
		11,445,169	C/A	substitution	Exon11		
		11,445,144	C/G	substitution	Exon11		
		11,445,126	G/T	substitution	Exon11		
T1	A06	20,874,321	G/A	substitution	Exon2	CT001_A06231480	PREDICTED: probable inorganic phosphate transporter 1-3 [Brassica rapa]
		20,874,445	C/T	substitution	Exon2		
		20,874,447	T/G	substitution	Exon2		
T3	A08	9,326,072	A/T	substitution	Exon4	CT001_A08282960	PREDICTED: S-type anion channel SLAH2 [Brassica rapa]
		9,326,066	C/T	substitution	Exon4		
		9,326,061	C/G	substitution	Exon4		
		9,326,048	G/C	substitution	Exon4		
		9,326,047	G/C	substitution	Exon4		
		9,326,043	A/G	substitution	Exon4		
		9,326,041	G/C	substitution	Exon4		
		9,326,040	G/C	substitution	Exon4		
		9,325,993	G/A	substitution	Exon4		
H1	A01	6,041,590	C/T	substitution	Exon1	CT001_A01012460	PREDICTED: RING-H2 finger protein ATL81 [Brassica rapa]
H2	A06	2,937,545	T/C	substitution	Exon7	CT001_A06202820	kinesin-like protein KIN-UA [Brassica napus]
S1	A03	4,844,784	T/A	substitution	Exon2	CT001_A03085970	PREDICTED: protein VERNALIZATION INSENSITIVE 3-like [Brassica rapa]
S2	A03	20,448,851	C/T	substitution	Exon1	CT001_A03117730	PREDICTED: heat shock factor-binding protein 1-like [Brassica rapa]
S3	A03	21,569,455	A/AT	insertion	Exon3	CT001_A03119840	PREDICTED: abscisic acid 8′-hydroxylase 1 [Brassica rapa]
		21,569,458	AC/A	deletion	Exon3		
S4	A07	15,357,787	C/T	substitution	Exon4	CT001_A07256220	PREDICTED: LOW QUALITY PROTEIN: chaperone protein ClpB1-like [Brassica rapa]
S5	A07	17,476,525	C/T	substitution	Exon5	CT001_A07260320	calmodulin-binding transcription activator 4-like isoform X2 [Brassica napus]
		17,476,532	G/C	substitution	Exon5		
S6	A10	306,428	C/T	substitution	Exon2	CT001_A10358000	uncharacterized protein LOC18027100 [Eutrema salsugineum]

^Z^: Chromosome number.

## Data Availability

The datasets generated and/or analyzed during the current study are available from the corresponding author on reasonable request.

## Supplementary Materials

The following are available online at https://www.mdpi.com/article/10.3390/genes12050621/s1, Table S1: List of primer sets for the exonic SNP candidates identified in transgenic Chinese cabbage plants, Table S2: Raw and trimmed resequencing data of the non-transgenic and transgenic Chinese cabbage plants, Table S3: Mapping results of non-transgenic and transgenic Chinese cabbage plant group.
