# Supplementary material for: Identification and Validation of Genetic Variations in Transgenic Chinese Cabbage Plants (Brassica rapa ssp. pekinensis) by Next-Generation Sequencing
Source: Genes (Basel). 2021 Apr 22;12(5):621. doi: 10.3390/genes12050621 (PMC8143544; doi:10.3390/genes12050621)
Supplement: Supplementary file 1 [file genes-12-00621-s001.zip › genes-1188767-supplementary.pdf]

Table S1. List of primer sets for the exonic SNP candidates identified in transgenic Chinese cabbage plants.

Name	Target gene with variation	Primer	Sequence (5'→3')	Expected product size
K3	CT001_A03120110	F	GAG GAA GTA GCA ATC TGT	183 bp
		R	GAA CTC GTG ACA TCA ATG	
G1	CT001_A03403810	F	GCT GAT TCA GGT TAT CTC TCG	180 bp
		R	CCA TAA CCA TAC AGA CTC GC	
T1	CT001_A03130860	F	CTT CTA TCA CCA GAA CTA G	180 bp
		R	CGG GTA GTA ATG ATC CAG	
T3	CT001_A06415940	F	CTC TCT GCT GTT GGG GTT G	144 bp
		R	GCC ATA TAG CGA GGG ATT GC	
H1	CT001_A01012460	F	CTG GTA CTT TCA GGT CTT AG	227 bp
		R	GAT AGA GGA ACA TGC TCA TC	
H2	CT001_A06202820	F	GGA AGC CAA ATC TAT CAA TCT C	190 bp
		R	TGT CTC TCC TCG GTG TCG T	
S1	CT001_A03085970	F	GAT GAA GAT GGC TCG TGG TA	120 bp
		R	GAA ACA GAT TCT GAT GGT GG	
S2	CT001_A03117730	F	AGA ATC AAT GAG CTG GAG C	105 bp
		R	CGG TGT TTT GGG TTC ATC GC	
S3	CT001_A03119840	F	GCT ATG AAG GCT CGC AAG GA	135 bp
		R	GAT CTG TTC GTC GCT CAG C	
S4	CT001_A07256220	F	CGA GAA GGA CAG GTT GAT TG	138 bp
		R	GAT TCA GTT GGC TGT TGT GG	
S5	CT001_A07260320	F	CCT ACT CGT TAT TCT GAG C	165 bp
		R	CAC CTT TGT AGT TTC ATT CG	
S6	CT001_A10358000	F	GGC TTT AAT CTC TTC AACA TC	157 bp
		R	TCA CAA CCA ACT TCC AAC CC	

Table S2. Raw and trimmed resequencing data of the non-transgenic and transgenic Chinese cabbage plants

	Name	Total raw reads	Total raw bases	Trimmed reads	Trimmed bases
Control	CT001	34,332,178	5,163,324,609	31,702,960	4,321,143,630
T_1	IGA7	34,639,750	5,209,327,231	31,625,346	4,331,305,874
T_3	IGA743	34,181,022	5,140,452,962	31,337,864	4,278,608,798

Table S3. Summary statistics for mapping evaluation of non-transgenic and transgenic Chinese cabbage plants.

	Name	Total reads count	Total reads length (bp)	Mapped reads count	Mapped reads length (bp)	Properly mapped paired reads	Average depth	Genome coverage (%)
Control	CT001	23,954,084	3,066,896,132	20,672,865	2,460,619,929	15,708,992	11.20×	98.00
T_1	IGA7	24,194,706	3,131,596,634	20,947,186	2,520,494,387	15,726,260	11.47×	98.02
T_3	IGA743	23,672,400	3,032,614,568	20,369,533	2,435,187,250	15,307,950	11.08×	97.45
